# Supplementary material for: Inorganic perovskite-based active multifunctional integrated photonic devices
Source: Nat Commun. 2024 Feb 20;15:1536. doi: 10.1038/s41467-024-45565-9 (PMC10879536; doi:10.1038/s41467-024-45565-9)
Supplement: Supplementary file 1 — Supplementary Information [file 41467_2024_45565_MOESM1_ESM.pdf]

Supplementary Information for

Inorganic Perovskite-Based Active Multifunctional Integrated Photonic Devices

This PDF file includes:

Part 1:

Supplementary Figures S1 to S16.

Part 2:

Introduction about the basic function of the fabricated photonic devices.

Part 3:

Supplementary References.

Part 1

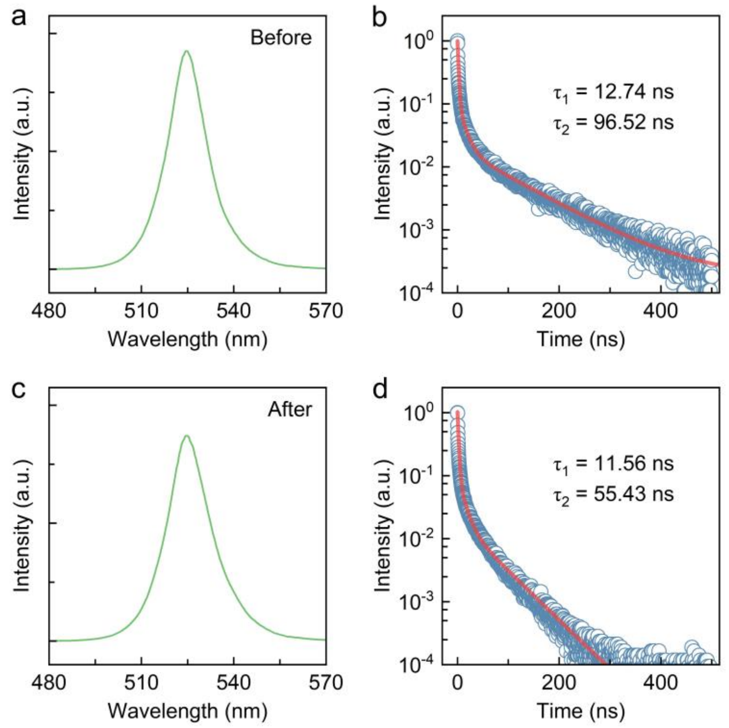

Supplementary Fig. S1 | (a-d) Room-temperature photoluminescence (PL) emission and Time-resolved PL delay dynamics contrast of the monocrystalline $CsPbBr_3$ thin film ((a) and (b), before focused ion beam (FIB) treatment) and microstructure ((c) and (d), after FIB treatment), showing the nearly consistent intensity and shape and demonstrating the feasibility of directly fabricating photonic devices.

In order to assess the extent of the damage caused by focused ion beam (FIB) etching, we characterized the photoluminescence (PL) emission and time-resolved PL (TRPL) delay dynamics of the monocrystalline $CsPbBr_3$ thin film and microstructure (after FIB treatment), respectively, as shown in Fig. S1.

The nearly consistent shape and strength of the PL emission before and after FIB treatment demonstrate the feasibility of fabricating photonic devices directly (Fig. S1a and c). As for the TRPL lifetime, one can observe the two shorter different time scales which are attributed to the radiative recombination of intrinsic excitons (slow) and the

bimolecular recombination process of excitons (fast) after FIB treatment, indicating more carrier trap defects are formed under higher ion-dose irradiation (Fig. S1b and d)[1-3]. It should be noted that our active integrated photonic devices operate in the lasing region (coherent emission), and then utilize such generated microlaser to realize the manipulation of waveguide coupling and propagation. Therefore, such a small number of carrier trap defects has little impact on the function of the devices and the concept we proposed.

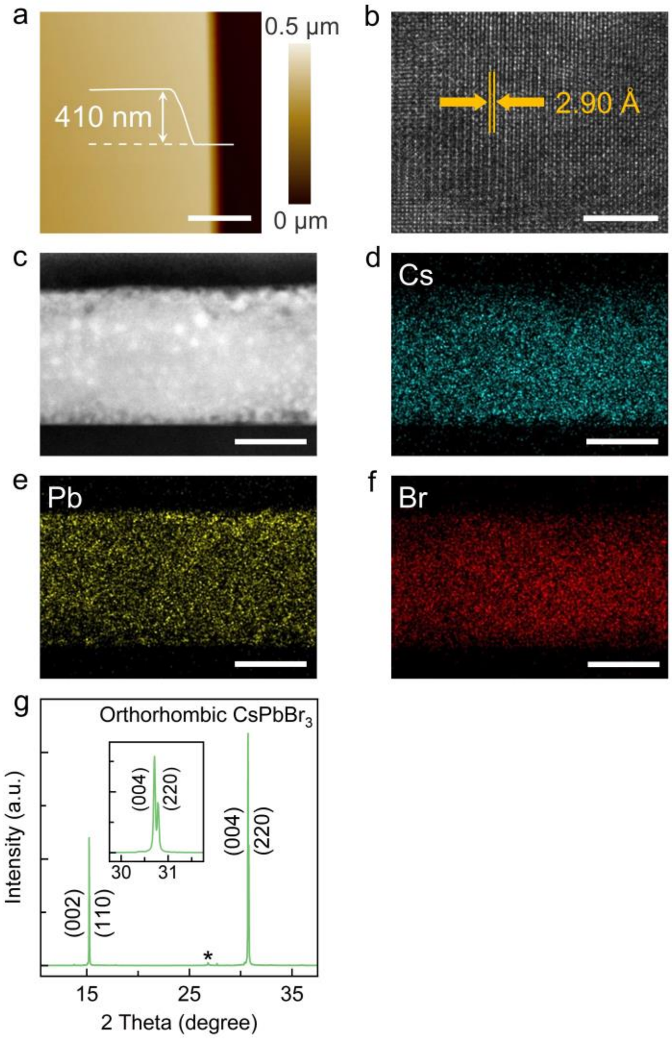

Supplementary Fig. S2 | (a) Atomic force microscopy image of a monocrystalline $CsPbBr_3$ thin film, showing a typical thickness of 410 nm. Scale bar: 1 μm. **(b)** High-resolution transmission electron microscopy (TEM) image of the monocrystalline $CsPbBr_3$ thin film, demonstrating the lattice spacing[4,5] of 0.29 nm. Scale bar: 5 nm. **(c-f)** Cross-sectional TEM image and energy-dispersive X-ray spectrometry elemental mappings of the monocrystalline $CsPbBr_3$ thin film, revealing a uniform spatial distribution of Cs, Pb, and Br elements. Scale bar: 50 nm. (g) Micro-region (a range of 200 micrometers) XRD of a monocrystalline $CsPbBr_3$ thin film, showing the orthorhombic phase structure[4,6] and good monocrystalline property. The XRD peak originating from the pure mica substrate is marked by *. The inset shows the magnified tiny splitting XRD peaks of the sample at ~ 30.7°.

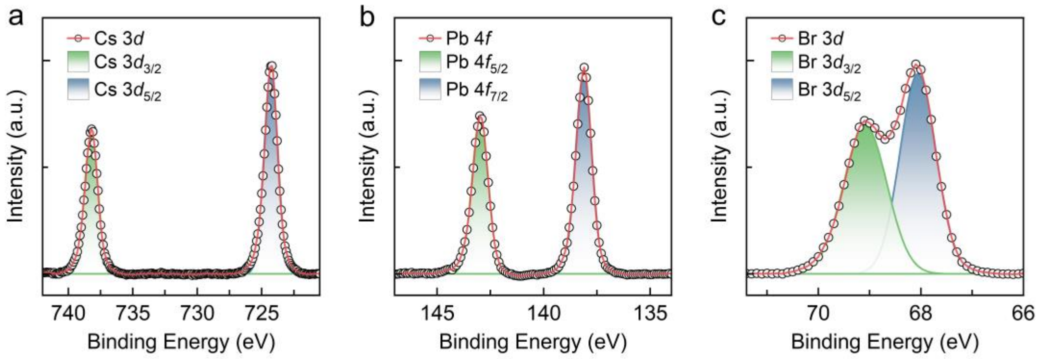

Supplementary Fig. S3 | (a-c) XPS analysis of the monocrystalline $CsPbBr_3$ thin film for Cs_{3d} (a), Pb_{4f} (b), and Br_{3d} (c), respectively. The presence of Cs, Pb and Br elements is consistent with the material composition of $CsPbBr_3$.

To further analyze the surface elemental composition and chemical state, we performed X-ray photoelectron spectroscopy (XPS) measurement on the monocrystalline $CsPbBr_3$ thin film. The XPS peaks of single Cs_{3d}, Pb_{4f}, and Br_{3d} are collected as shown in Fig. S3. Two peaks at around 738.2 eV and 724.3 eV can be assigned to Cs $3d_{3/2}$ and Cs $3d_{5/2}$, while the strong peaks located at 143.0 eV and 138.1 eV are attributed to Pb $4f_{5/2}$ and Pb $4f_{7/2}$, respectively. The prominent peaks of Br $3d_{3/2}$ and Br $3d_{5/2}$ of Br_{3d} are observed at around 69.1 eV and 68.0 eV. Meanwhile, analysis of the presence of Cs, Pb, and Br elements is nearly consistent with the material composition of $CsPbBr_3$, demonstrating the high chemical purity of the $CsPbBr_3$ thin film on mica through direct CVD synthesis.

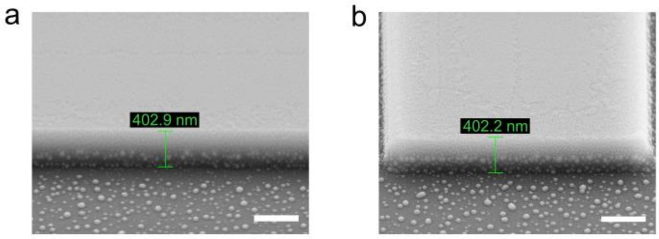

Supplementary Fig. S4 | Scanning electron microscopy (SEM) images of the $CsPbBr_3$ microwire after FIB treatment, showing the smooth surface and sharp edges to form a transverse Fabry-Pérot (FP) microcavity[7-9] and a height of 525 nm. **(a)** Side facet. **(b)** End facet. Scale bar: 500 nm. Tilted by 50° with respect to the horizontal position for (a-b).

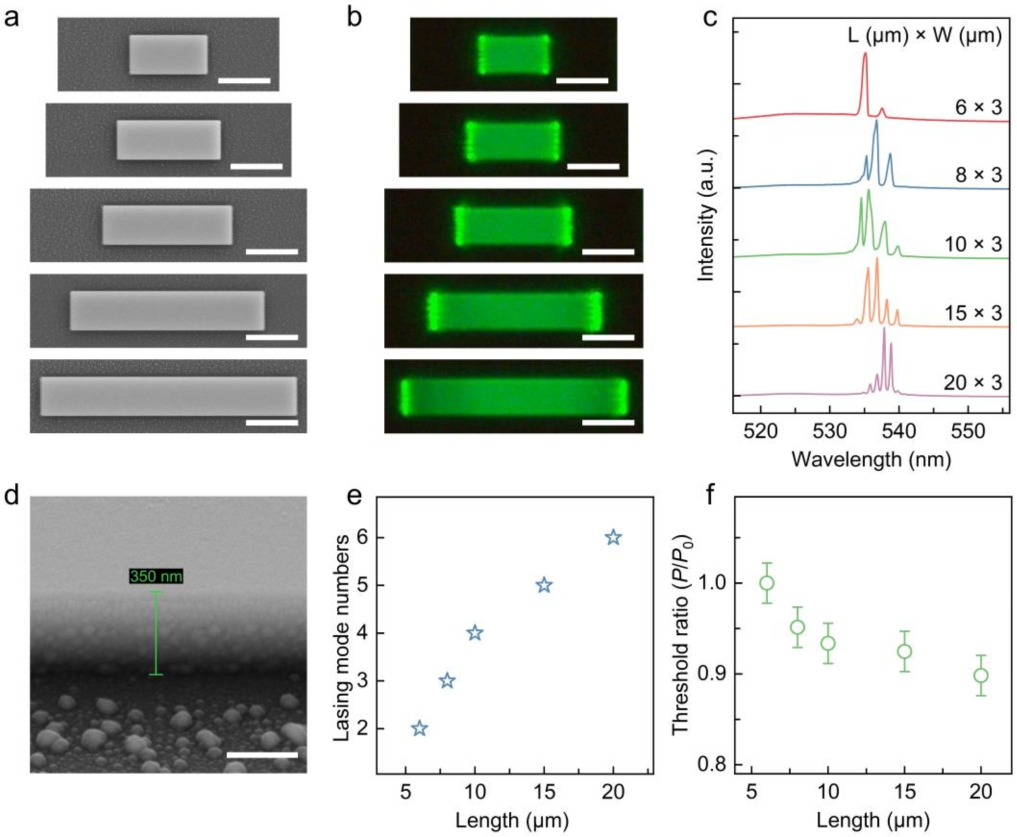

Supplementary Fig. S5 | (a) SEM images of $CsPbBr_3$ microwires with identical widths of 3 μm and different lengths from 6 μm to 20 μm. Scale bar: 4 μm. **(b)** Corresponding PL microscope images of the different-length microwires above the threshold. Scale bar: 4 μm. **(c)** The normalized lasing emission spectra of the different-length microwires above the threshold. **(d)** SEM image of the side facet for one microwire (tilted by 50° with respect to the horizontal position), indicating a height of 457 nm. Scale bar: 300 nm. Such microwires are of identical thickness. **(e)** Evolution of the lasing mode number as a function of the length of microwire. **(f)** Evolution of the lasing threshold as a function of the length of microwire, setting the threshold of microwire with the dimension of 6 μm × 3 μm as P_0.

To investigate the cavity geometry-dependent lasing performance, we have measured the lasing properties of the multiple different length $CsPbBr_3$ microwires, as shown in Fig. S5.

Figure S5a displays the top view SEM images of five etched different-length $CsPbBr_3$ microwires, with the dimensions of 6 μm × 3 μm, 8 μm × 3 μm, 10 μm × 3 μm, 15 μm

× 3 μm and 20 μm × 3 μm, respectively. All the microwires exhibit smooth surfaces and sharp edges with an identical thickness of 457 nm (Fig. S5d). Under the excitation by a 400 nm femtosecond pulsed laser above the threshold, the PL microscope images reveal that strong lasing emissions distinctly leak out from the opposite end facets of the microwires, which can be attributable to the FP mode oscillation (Fig. S5b). The normalized lasing emission spectra for the five different-length microwires are shown in Fig. S5c, demonstrating the evolution of the lasing mode number *versus* the length. The lasing mode numbers increase from 2 to 6 when the lengths of microwires change from 6 μm to 20 μm (Fig. S5e). Thus, we can precisely control the number of output lasing modes by adjusting the length of the FP microcavity. To compare the change of lasing threshold, we set the threshold for microwire with the dimension of 6 μm × 3 μm as P_0, and calculated the threshold ratio of these different-length microwires. One can see that lasing thresholds of perovskite microwires exhibit a decay trend with the length increasing, which could be attributed to the enlarged gain medium region (Fig. S5f).

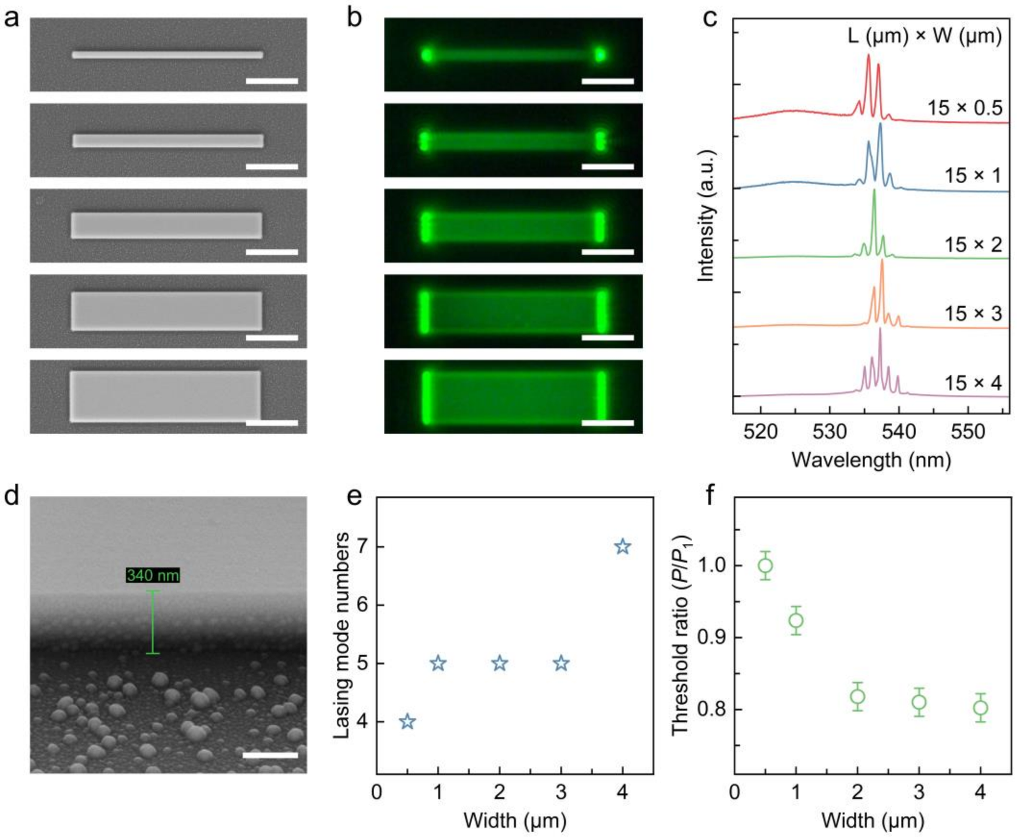

Supplementary Fig. S6 | (a) SEM images of $CsPbBr_3$ microwires with identical lengths of 15 μm and different widths from 0.5 μm to 4 μm. Scale bar: 4 μm. **(b)** Corresponding PL microscope images of the different-width microwires above the threshold. Scale bar: 4 μm. **(c)** The normalized lasing emission spectra of the different-width microwires above the threshold. **(d)** SEM image of the side facet for one microwire (tilted by 50° with respect to the horizontal position), indicating a height of 444 nm. Scale bar: 300 nm. Such microwires are of identical thickness. **(e)** Evolution of the lasing mode number as a function of the width of microwire. **(f)** Evolution of the lasing threshold as a function of the width of microwire setting the threshold of microwire with the dimension of 15 μm × 0.5 μm as P_1.

To investigate the cavity geometry-dependent lasing performance, we have measured the lasing properties of the multiple different width $CsPbBr_3$ microwires, as shown in Fig. S6.

Figure S6a shows the top view SEM images of five different-width microwires, with the dimensions of 15 μm × 0.5 μm, 15 μm × 1 μm, 15 μm × 2 μm, 15 μm × 3 μm and

15 μm × 4 μm, respectively. All the microwires process smooth surfaces and sharp edges with an identical thickness of 444 nm (Fig. S6d). Excited by a 400 nm femtosecond pulsed laser above the threshold, one can see that such microwires display strong lasing emissions in the same type, which distinctly leak out from the opposite end facets and originate from the FP oscillation modes (Fig. S6b). Figure S6c displays the normalized lasing emission spectra for the five different-width microwires, demonstrating the evolution of the lasing mode number *versus* the width. Unlike the results of the different length microwires, due to the length limitation of the FP microcavity, the number of lasing modes is stable around 5 with the change of width (Fig. S6e). As for the variation trend of the lasing threshold, similarly, we set the threshold for microwire with the dimension of 15 μm × 0.5 μm as P_1, and calculated the threshold ratio of these different width microwires. The results also indicate that the thresholds of the perovskite microwires exhibit a decay trend with the width increasing, attributed to the enlarged gain medium region (Fig. S6f).

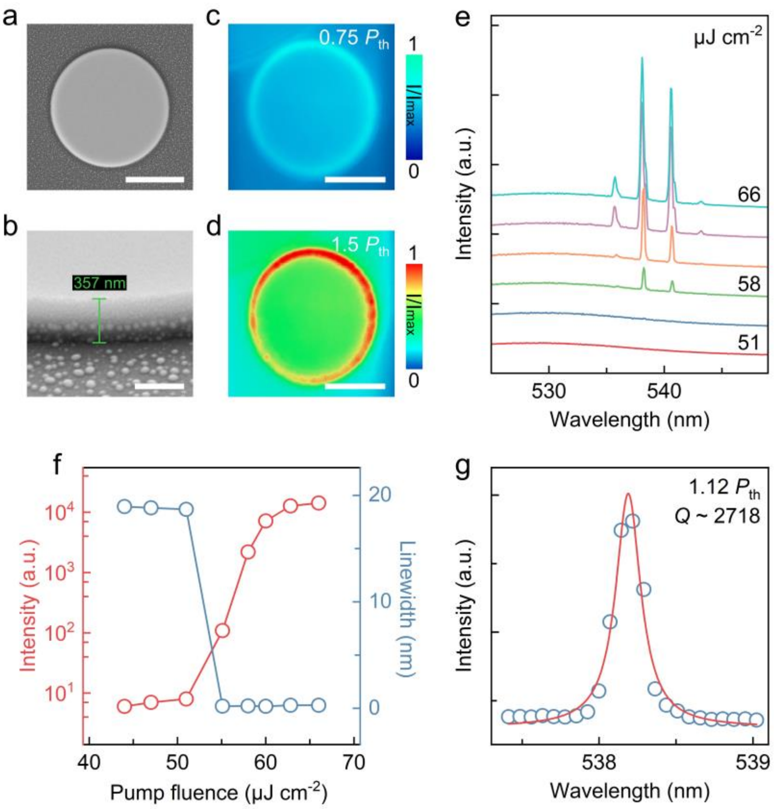

Supplementary Fig. S7 | (a) SEM image of a $CsPbBr_3$ microdisk with a diameter of 8 μm after FIB treatment. Scale bar: 4 μm. **(b)** SEM image of the side facet for the microdisk (tilted by 50° with respect to the horizontal position), indicating a height of 466 nm. Scale bar: 400 nm. **(c-d)** Corresponding real-space PL images of the microdisk below (c) and above (d) the threshold, respectively. Scale bar: 4 μm. (**e**) PL spectra emitted from the microdisk with the pump fluence increasing from 51 μJ cm^{-2} to 66 μJ cm^{-2}. **(f)** Evolution of the integrated PL emission intensity (red curve) and linewidth (blue curve) as functions of pump fluence of the microdisk, showing the threshold of 53.8 μJ cm^{-2}. **(g)** One magnified lasing oscillation mode with a Lorentz-fitted linewidth of 0.198 nm and a *Q*-factor of 2718.

An etched $CsPbBr_3$ microdisk with whispering gallery mode (WGM) for lasing has been still investigated to compare the effect of the cavity geometry on lasing oscillation modes, as shown in Fig. S7.

Figure S7a shows the top view SEM image of the obtained $CsPbBr_3$ microdisk after FIB treatment, where the disk has a smooth surface with a diameter of 8 μm. Also, the

smooth surface and sharp edges of the microdisk can be verified from the magnified SEM image, and the thickness is extracted as 466 nm (Fig. S7b). At room temperature, under low-power excitation by a 400 nm femtosecond pulsed laser, the whole disk presents a near-uniform PL emission (Fig. S7c). Under high pump fluence and above the threshold, strong PL emission appears around the disk, which is visibly distinguished from emission in the in-plane region and indicates the WGM mode laser arises (Fig. S7d).

Figure S7e displays the PL emission evolution with the pump fluence of the microdisk. One can see that with the pump fluence increasing from 51 μJ cm^{-2} to 66 μJ cm^{-2}, several sharp peaks of lasing emission emerge at the low-energy side of spontaneous emission, and become dominant in the PL spectra with the intensity rapidly rise. A nonlinear process of the lasing emission in the microdisk is revealed by the evolution of the integrated PL intensity and linewidth as functions of pump fluence (Fig. S7f). The dramatical decrease of the linewidth and the typical S-shaped growth curve of the PL intensity unambiguously indicate the arising of lasing behavior with the pump fluence increasing. Here, the critical threshold of the microdisk is extracted as 53.8 μJ cm^{-2}. Figure S7g illustrates the Lorentz fitting of one magnified lasing oscillation mode with a linewidth of 0.198 nm and a Q-factor of 2718 at 1.12 P_{th}, indicating the high lasing performance of the etched perovskite microdisk.

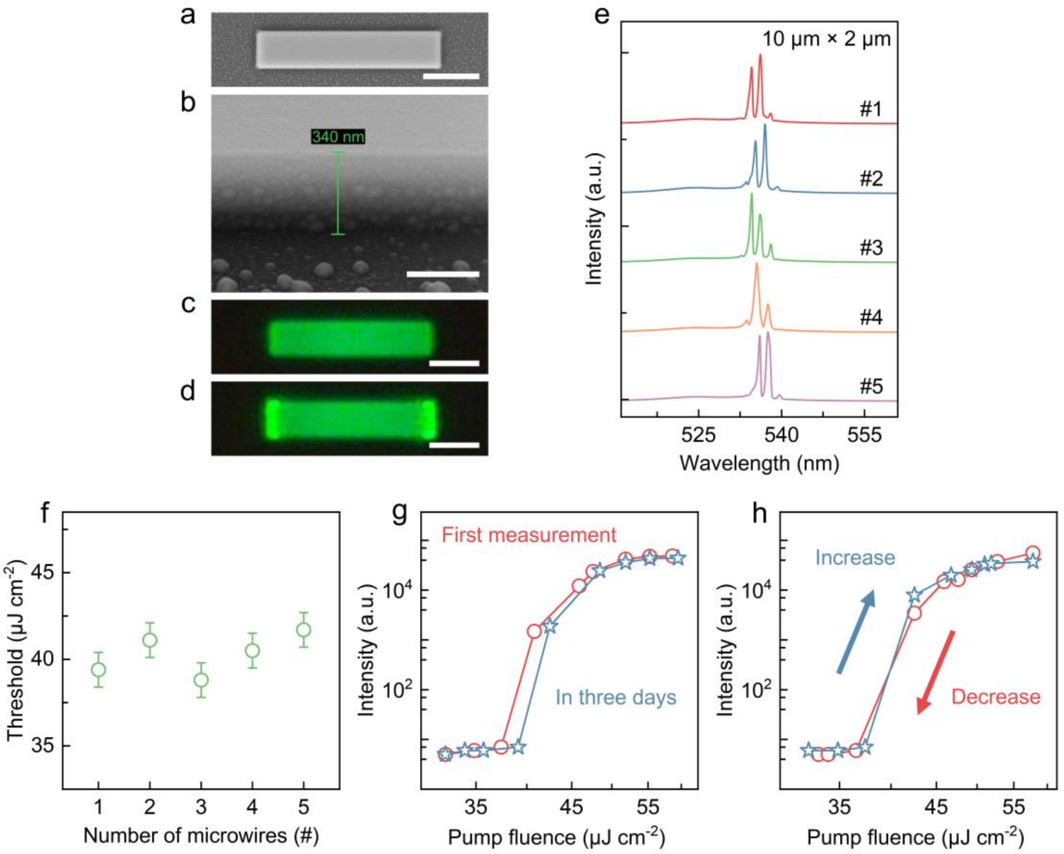

Supplementary Fig. S8 | (a) SEM image of a $CsPbBr_3$ microwire with a dimension of 10 μm × 2 μm. Scale bar: 3 μm. **(b)** SEM image of the side facet for the microwire (tilted by 50° with respect to the horizontal position), indicating a height of 444 nm. Scale bar: 300 nm. **(c, d)** Corresponding PL microscope images of the microwire below (c) and above (d) the threshold, respectively. Scale bar: 3 μm. **(e)** Normalized lasing emission spectra for five $CsPbBr_3$ microwires of identical dimension, showing near-unanimous peak shape and number of lasing modes. **(f)** Statistics of the lasing threshold for the five microwires. **(g, h)** Integral emission intensity as a function of pump fluence under different times and excitation sequences.

The optical stability of microwire lasers is crucial for the efficient operation of integrated photonic devices. We measured the lasing properties of five $CsPbBr_3$ microwires of the same size to investigate the stability and uniformity of the designed laser devices, as shown in Fig. S8.

As shown in Fig. S8a and b, a typical etched $CsPbBr_3$ microwire exhibits a smooth surface and sharp edges with a dimension of 10 μm × 2 μm × 0.444 μm. Under low-power excitation by a 400 nm femtosecond pulsed laser, the PL microscope image of the microwire reveals a uniform green-color emission (Fig. S8c). When the pump fluence increases above the threshold, strong lasing emission is observed to distinctly leak out from the opposite end facets of the microwire, owing to the FP mode oscillation (Fig. S8d). The normalized lasing emission spectra for the five microwires of identical dimensions are shown in Fig. S8e, demonstrating the near-unanimous peak shape and number of lasing modes. One can observe that under the same measurement configuration, the thresholds of these microwire lasers exhibit small fluctuation, which also indicates the uniformity of the designed laser devices (Fig. S8f).

Moreover, we performed measurements for the stability and robustness of microwire laser. The emission intensity as a function of pump fluence is measured a few days after the first measurement. One can observe that the emission intensity and threshold of lasing have hardly changed much, as shown in Fig. S8g. Furthermore, we measured the power-dependent emission intensity by repeatedly increasing and decreasing pumping power, and we still observed that the lasing did not degenerate, as shown in Fig. S8h. Thus, we believe the etched perovskite microwire laser is stable and robust under high optical excitation.

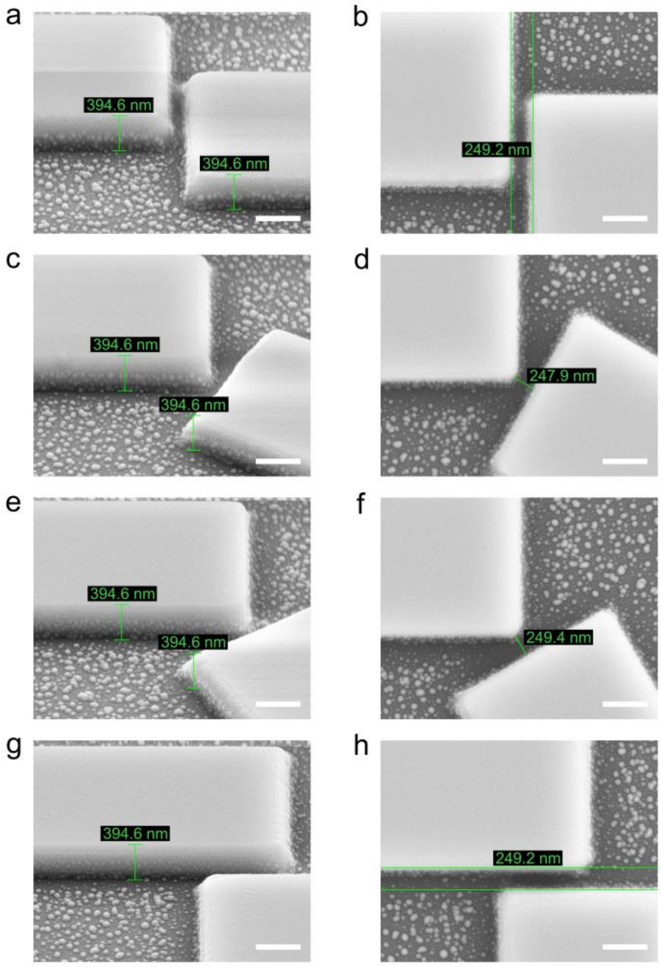

Supplementary Fig. S9 | SEM images of the coupling regions for the $CsPbBr_3$ microwire waveguide couplers at different coupling angles of 0° **(a)** and **(b)**, 30° **(c)** and **(d)**, 60° **(e)** and **(f)**, and 90° **(g)** and **(h)**, respectively, demonstrating the identical height of 515 nm and gaps of 250 nm to separate the microwires. Scale bar: 500 nm. Tilted by 50° with respect to the horizontal position for (a), (c), (e) and (g), and top view for (b), (d), (f) and (h).

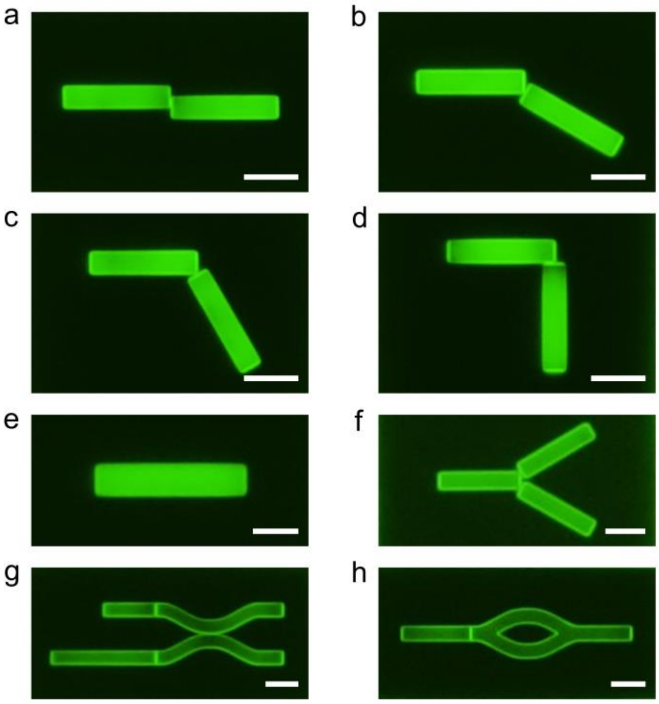

Supplementary Fig. S10 | Fluorescence microscope images of the $CsPbBr_3$ multifunctional photonic devices, showing uniform and strong fluorescence emission after FIB treatment. Microwire waveguide couplers at different coupling angles of 0° **(a)**, 30° **(b)**, 60° **(c)**, and 90° **(d)**, respectively. Scale bar: 5 μm. **(e)** Microwire laser. Scale bar: 3 μm. **(f-h)** Active integrated beam splitter, X-coupler and Mach-Zehnder interferometer (MZI), respectively. Scale bar: 5 μm.

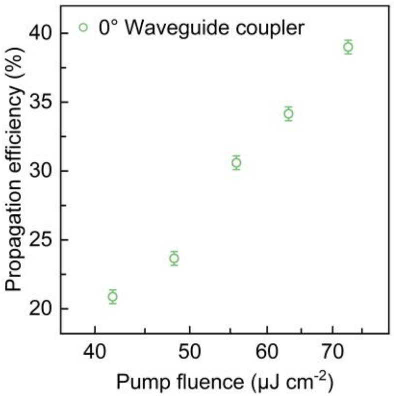

Supplementary Fig. S11 | The evolution of coupled lasing propagation efficiency as a function of pump fluence of the microwire waveguide coupler at coupling angle of 0° (with error bars originating from the fitting of lasing integral intensity), displaying a rising tendency from 20.88% to 39.00% with the pump fluence increasing.

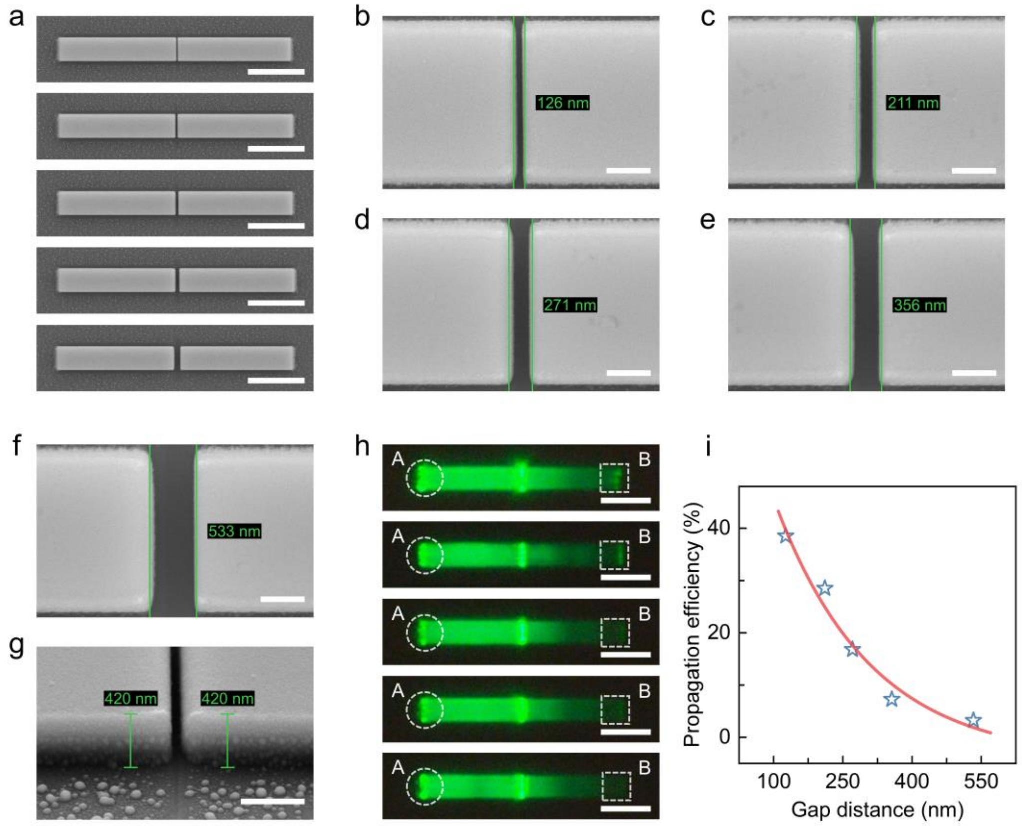

Supplementary Fig. S12 | (a) SEM images of $CsPbBr_3$ waveguide couplers with different gaps between two microwires. The microwires are of identical dimension (10 μm × 2 μm). Scale bar: 5 μm. **(b-f)** Magnified SEM images of the different gaps between two microwires, showing the gaps of 126 nm (b), 211 nm (c), 271 nm (d), 356 nm (e) and 533 nm (f). Scale bar: 500 nm. **(g)** SEM image of the side facet for one waveguide coupler (tilted by 50° with respect to the horizontal position), indicating a height of 548 nm. Scale bar: 500 nm. Such waveguide couplers are of identical thickness. **(h)** Lasing microscope images of the waveguide couplers with different gaps under the pump fluence of 62 μJ cm^{-2}. Scale bar: 5 μm. The white dashed circles and boxes depict excitation terminals and propagation terminals, respectively. **(i)** Evolution of coupled propagation efficiency for lasing as a function of the gap distance of microwire waveguide coupler, displaying a decay trend with the gap distance increasing.

To investigate the effect of the coupling gap on the coupled propagation efficiency for lasing, we performed lasing waveguide-coupling measurements between two etched $CsPbBr_3$ microwires separated by different coupling gaps, as shown in Fig. S12. The

result displays a decay trend of the propagation efficiency for lasing with the gap distance increasing.

The $CsPbBr_3$ waveguide couplers with different coupling gaps are composed of two microwires with identical dimensions of 10 μm × 2 μm × 0.548 μm (Fig. S12a and g). Meanwhile, the different coupling gaps between two microwires are confirmed of 126 nm, 211 nm, 271 nm, 356 nm and 533 nm from the magnified SEM images, respectively (Fig. S12b-f). In these configurations, one microwire served as the lasing source, while the other functioned as the propagation medium.

When excited above the threshold, the leftmost microwire emits lasing, and the propagating coupled signals are detected at the terminals of the waveguide coupler to compare the PL spectra of input and output. Figure S12h shows the lasing microscope images of the waveguide couplers with different coupling gaps under the pump fluence of 62 $\mu J\ cm^{-2}$. The observed transition from light to dark of the propagation terminals reveals the intensity of lasing propagation degenerates with the gap distance increasing. Analysis of the propagation efficiency for lasing indicates a decay trend from 38.53% to 3.23% when the gap distance increases from 126 nm to 533 nm (Fig. S12i). Therefore, a smaller coupling gap can lead to improved performance of coupled photonic devices in practical applications.

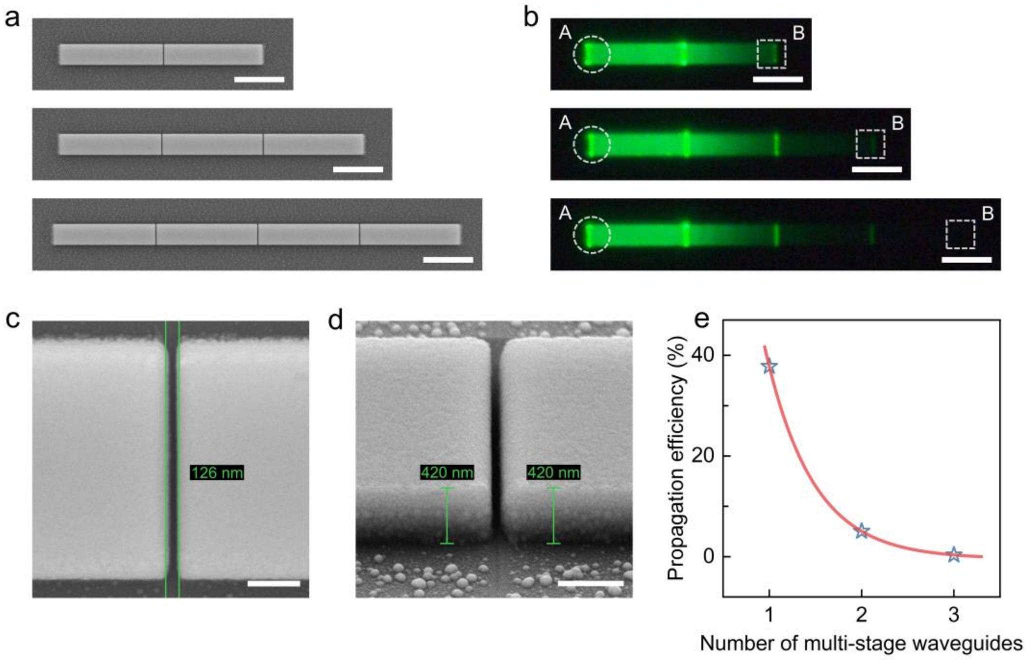

Supplementary Fig. S13 | (a) SEM images of $CsPbBr_3$ multi-stage waveguide couplers with identical gaps between microwires. The microwires are of identical dimension (10 μm × 2 μm). Scale bar: 5 μm. **(b)** Lasing microscope images of the multi-stage waveguide couplers under the pump fluence of 62 μJ cm^{-2}. Scale bar: 5 μm. The white dashed circles and boxes depict excitation terminals and propagation terminals, respectively. **(c)** Magnified SEM image of the gap between two microwires, showing the gap of 126 nm. Scale bar: 500 nm. **(d)** SEM image of the side facet for one multi-stage waveguide coupler (tilted by 50° with respect to the horizontal position), indicating a height of 548 nm. Scale bar: 500 nm. Such multi-stage waveguide couplers are of identical thickness. **(e)** Evolution of coupled propagation efficiency for lasing as a function of the number of multi-stage waveguides at 62 μJ cm^{-2}, displaying a decay trend with the stage increasing.

To investigate the lasing coupling efficiency of multi-stage waveguides, we performed multi-stage lasing waveguide-coupling measurements between etched $CsPbBr_3$ microwires separated by identical coupling gaps, as shown in Fig. S13. The result displays a decay trend of the propagation efficiency for lasing *versus* the number of multi-stage waveguides.

The morphologies of $CsPbBr_3$ multi-stage waveguide couplers are exhibited in Fig. S13a, with the stages from 1 to 3, respectively. All the waveguide couplers are separated by identical coupling gaps of 126 nm and composed of microwires with identical dimensions of 10 μm × 2 μm × 0.548 μm (Fig. S13a, c and d). In these configurations, one microwire served as the lasing source, and the others functioned as the propagation medium.

When excited above the threshold, the leftmost microwire emits lasing, and the propagating coupled signals are detected at the terminals of the waveguide coupler to compare the PL spectra of input and output. Figure S13b shows the lasing microscope images of the multi-stage waveguide couplers under the pump fluence of 62 μJ cm^{-2}, and the observed transition from light to dark of the propagation terminals reveals the intensity of lasing propagation is degenerate with stage increasing. Meanwhile, analysis of the propagation efficiency for lasing indicates a decay trend from 37.8% to 0.35% corresponding to the stage from 1 to 3 (Fig. S13e).

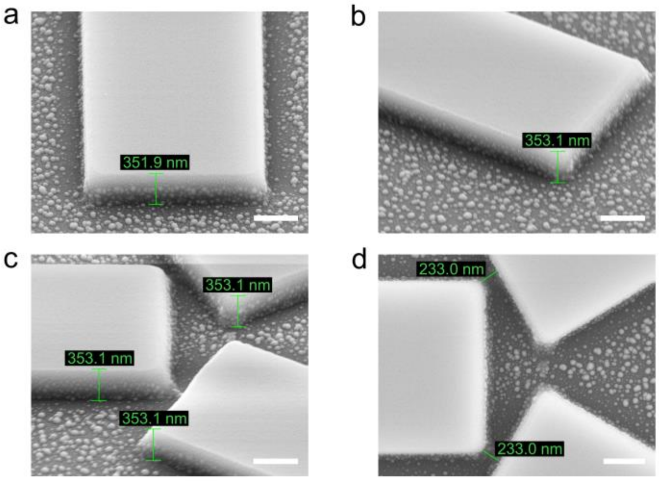

Supplementary Fig. S14 | SEM images of the $CsPbBr_3$ active integrated beam splitter, demonstrating the identical height of 460 nm and gaps of 233 nm to separate the microwires. **(a-b)** End facets of the microwire laser and the lower coupled microwire propagation arm, respectively. **(c-d)** Coupling region and subwavelength gaps of the beam splitter. Scale bar: 500 nm. Tilted by 50° with respect to the horizontal position for (a-c), and top view for (d).

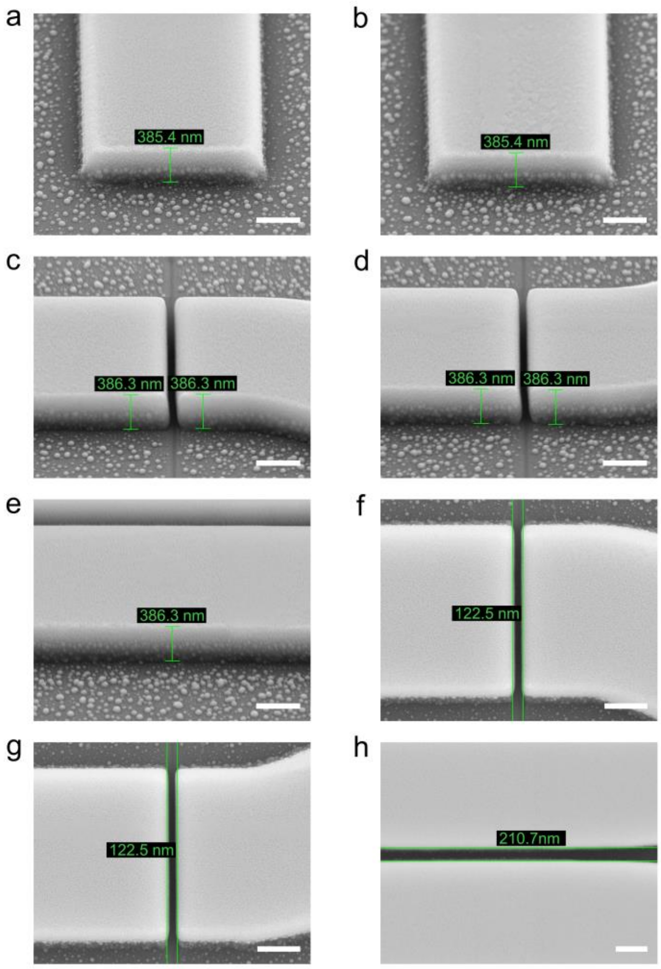

Supplementary Fig. S15 | SEM images of the $CsPbBr_3$ active integrated X-coupler, showing the identical height of 500 nm, and gaps of 122.5 nm and 210.7 nm to separate the source and two curved waveguides. **(a-b)** End facets of the two microwire lasers. **(c-h)** Coupling regions and the subwavelength gaps between the source and device, and the two curved waveguides, respectively. Scale bar: 500 nm. Tilted by 50° with respect to the horizontal position for (a-e), and top view for (f-h).

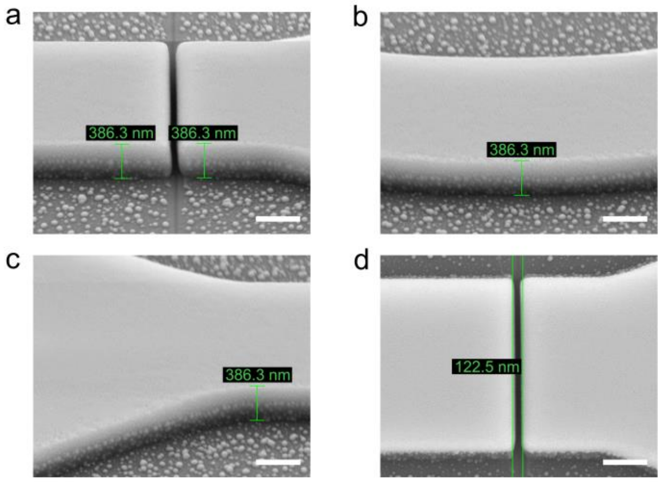

Supplementary Fig. S16 | SEM images of the $CsPbBr_3$ active integrated MZI, demonstrating the identical height of 500 nm and a gap of 122.5 nm to separate the source and device. **(a-c)** Coupling region, the lower waveguide arm and coalescence region for the MZI, respectively. **(d)** The subwavelength gap between source and device. Scale bar: 500 nm. Tilted by 50° with respect to the horizontal position for (a-c), and top view for (d).

Part 2

The basic functions of the fabricated photonic devices are described below:

Micro beam splitter: As a basic component in the integrated optical path, the Y-type beam splitter has a very simple structure and its main function is to realize the beam splitting and coalescence of light. An optical field (light), incident in the input port of the waveguide, is divided into two beams by the Y-branch structure and can be detected at the output end coherently. The beam splitting ratio of the Y branch is defined as P_{out1} / P_{out2}. The design of the Y-type beam splitter should meet the following requirements: a high propagation efficiency, a stable splitting ratio and a small device size.

Micro X-Coupler: X-Coupler is a basic component used in many kinds of photonic circuits. Cross-waveguide coupling can occur when two waveguides are brought close together and interact *via* the evanescent fields outside their boundaries. A typical X-coupler is a four-port device with two input and two output ports. In such structure, an optical field (light), incident in one of the input ports, can be split coherently into two parts through the waveguides and coupling gap and finally detected at the two output ports, respectively. Following this strategy, a transfer matrix extracted from the individual two input and two output ports can be given, as described in the equation in Figure 1b of the main text.

Mach-Zehnder interferometer (MZI): MZI is an optical device based on the principle of light interference, which is widely used to modulate the intensity and phase of output signals in fields such as optical communication, lidar, optical sensors, and so on. In a typical MZI device, the optical structure can guide the propagation direction of input light. The light signals simultaneously split along the two arms of the device respectively, and finally coalesce and interfere at the output end. By modulating the optical path difference between the two arms, the interference phase of the output signal can be tuned.

For the perovskite-based MZI in our work, one of the modulation potentials in future applications of MZI devices is the introduction of an electrical structure, *e.g.*, applying a voltage to one arm of the device, to change the refractive index of materials and optical length, which can be used to modulate the phase and intensity of light propagation. After the coalescence of the beams from the two arms, the phase change caused by the electrical structure modulation will be transformed into the light intensity change of the output light signals, to realize the signal modulation at the output end. In future work, various hybrid optoelectronic structures can be integrated with the perovskite-based active MZI to broaden its applications.

Part 3

Supplementary References

[1] S. Manser, J., V. Kamat, P. Band filling with free charge carriers in organometal halide perovskites. *Nat. Photonics* **8**, 737-743 (2014).

[2] Xing, G. et al. Transcending the slow bimolecular recombination in lead-halide perovskites for electroluminescence. *Nat. Commun.* **8**, 14558 (2017).

[3] Wang, Y. et al. Perovskite-ion beam interactions: toward controllable light emission and lasing. *ACS Appl. Mater. Interfaces* **11**, 15756-15763 (2019).

[4] Zhong, Y. et al. Large-scale thin $CsPbBr_3$ single-crystal film grown on sapphire *via* chemical vapor deposition: toward laser array application. *ACS Nano* **14**, 15605-15615 (2020).

[5] Wang, Y. et al. Large-area synthesis and patterning of all-Inorganic lead halide perovskite thin films and heterostructures. *Nano Lett.* **21**, 1454-1460 (2021).

[6] Zhao, L. et al. Vapor-phase incommensurate heteroepitaxy of oriented single-crystal $CsPbBr_3$ on GaN: toward integrated optoelectronic applications. *ACS Nano* **13**, 10085-10094 (2019).

[7] Zhu, H, et al. Lead halide perovskite nanowire lasers with low lasing thresholds and high quality factors. *Nat. Mater.* **14**, 636-642 (2015).

[8] Zhou, H. et al. Vapor growth and tunable lasing of band gap engineered cesium lead halide perovskite micro/nanorods with triangular cross section. *ACS Nano* **11**, 1189-1195 (2017).

[9] Eaton, S. W. et al. Lasing in robust cesium lead halide perovskite nanowires. *Proc. Natl. Acad. Sci. U.S.A* **113**, 1993-1998 (2016).
